# Natural Non-Mulberry Silk Nanoparticles for Potential-Controlled Drug Release

**DOI:** 10.3390/ijms17122012

**Published:** 2016-12-01

**Authors:** Juan Wang, Zhuping Yin, Xiang Xue, Subhas C. Kundu, Xiumei Mo, Shenzhou Lu

**Affiliations:** 1 National Engineering Laboratory for Modern Silk, College of Textile and Clothing Engineering, Soochow University, Suzhou 215123, China; juanwang1006@126.com (J.W.); 20154215016@stu.suda.edu.cn (Z.Y.); 20145215028@stu.suda.edu.cn (X.X.); 2 College of Chemistry and Chemical Engineering and Biological Engineering, Donghua University, Shanghai 201620, China; xmm@dhu.edu.cn; 3 Department of Biotechnology, Indian Institute of Technology Kharagpur, West Bengal 721302, India; kundu@hijli.iitkgp.ernet.in

**Keywords:** *Antheraea pernyi* silk fibroin, nanoparticles, controlled drug release

## Abstract

Natural silk protein nanoparticles are a promising biomaterial for drug delivery due to their pleiotropic properties, including biocompatibility, high bioavailability, and biodegradability. Chinese oak tasar *Antheraea pernyi* silk fibroin (*Ap*F) nanoparticles are easily obtained using cations as reagents under mild conditions. The mild conditions are potentially advantageous for the encapsulation of sensitive drugs and therapeutic molecules. In the present study, silk fibroin protein nanoparticles are loaded with differently-charged small-molecule drugs, such as doxorubicin hydrochloride, ibuprofen, and ibuprofen-Na, by simple absorption based on electrostatic interactions. The structure, morphology and biocompatibility of the silk nanoparticles in vitro are investigated. In vitro release of the drugs from the nanoparticles depends on charge-charge interactions between the drugs and the nanoparticles. The release behavior of the compounds from the nanoparticles demonstrates that positively-charged molecules are released in a more prolonged or sustained manner. Cell viability studies with L929 demonstrated that the *Ap*F nanoparticles significantly promoted cell growth. The results suggest that Chinese oak tasar *Antheraea pernyi* silk fibroin nanoparticles can be used as an alternative matrix for drug carrying and controlled release in diverse biomedical applications.

## 1. Introduction

Various applications in pharmaceutical and biomedical technology are based on the dispersion of particulates, which include specialty coatings and sustained release and delivery systems [1,2,3]. Interest is growing in the development of different drug delivery systems to meet the requirements of different diseases. Various synthetic and bio-polymers have been investigated to produce particulate carriers for sustained release [4,5,6,7,8]. However, the production of particles remains challenging because choosing appropriate materials and modes of processing requires avoiding surfactants, initiators, or organic solvents as far as practicable [9]. Natural macromolecule materials such as collagen, gelatin, and albumin are often preferred [10,11], which can be processed under mild conditions. Natural silk proteins are now considered a suitable material for drug delivery applications because of several important properties, such as biodegradation [12,13,14], biocompatibility [15,16,17], aqueous-based ambient purification [18], and effective drug stabilization [19,20]. Silk protein fibroin derived from wild silkworm sources is termed non-mulberry silk. Non-mulberry silks in different forms or matrices provide a range of superior natural biomaterials [21]. Silk protein is composed of diverse amino acids, many of which contain functional groups which can be used to bind to cell surface receptors of specific cell types. This binding is an advantage for the delivery of drugs and compares favorably with many other synthetic polymeric systems. Compared with *Bombyx mori* silk protein fibroin, *A. pernyi* silk fibroin (*Ap*F) is rich in Ala, Asp, and Arg, and has less Gly. In addition, *Ap*F contains the RGD (Arg-Gly-Asp) tripeptide sequence [22,23], which serves as a suitable receptor for and increases the binding affinity of cell surface receptors [24]. It is reported that *A. pernyi* silk fibroin provides much stronger cell adhesion compared to *Bombyx mori* and collagen [25]. For drug delivery, especially protein drugs, silk materials exhibit high encapsulation efficiency and controllable drug release kinetic [26,27]. In addition, silk particles are already exploited as a delivery vehicle for growth factors and anti-cancer therapeutics [28,29,30,31]. Furthermore, the high surface area of ultrafine silk particles increases the loading of the target molecules [32]. Hence, selection of natural silk particles as a platform is justified for controlled drug delivery.

There are several techniques available for the preparation of drug-loaded silk particles, such as self-assembly [33], layer-by-layer (LBL) deposition [34], emulsion-solvent evaporation spray drying [35], and phase separation [36]. However, each method has two-sidedness—advantages and disadvantages—so that it is important to choose an appropriate method in producing silk particles for drug delivery applications. Therefore, a more available preparation method is still needed for the formation of nanoparticles. Notably, the formulation of nanoparticles via ionic induction is gaining immense popularity. Silk particles have already been fabricated from an aqueous protein solution by the addition of ions [28,37]. However, the literature reporting non-mulberry *A. pernyi* silk microparticles and nanoparticles [38,39,40] as a suitable delivery vehicle is notably limited. Hence, the non-mulberry natural silk fibroin nanoparticle will be a viable platform for a controlled drug delivery system. It is reported that the particles of *A. pernyi* silk fibroin can also stabilize and deliver enzymes, such as lysozyme [41]. However, it is still underutilized as a biomaterial for regenerative medicine, even in the presence of RGD. One of the major challenges in the fabrication methods is the requirement of organic solvents, crosslinking agents, or initiators [9], which may cause damage to the human body.

In the present study, the *Ap*F nanoparticles are fabrication using cations (Ca^2+^) as reagents under mild conditions. Doxorubicin hydrochloride, ibuprofen, and ibuprofen-Na are selected as our positively-charged, uncharged, and negatively-charged model drugs to evaluate the controlled drug delivery profile. In addition, the morphology, size, surface area, zeta potential, the loading, loading efficiencies, and release kinetics in relation to charge of drug-loaded *Ap*F nanoparticles are investigated. In addition, the in vitro degradation and cell culture of the non-mulberry silk fibroin nanoparticles are also discussed. The results indicate that small-molecule drugs with different charges are suitable for sustained release from natural silk nanoparticles.

## 2. Results

### 2.1. Morphology of Drug-Loaded ApF Nanoparticles

**Figure 1:**
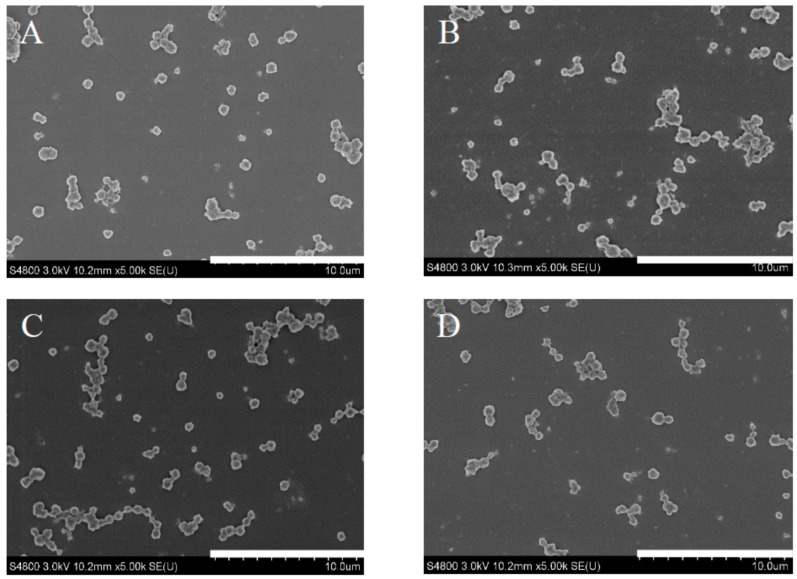
Scanning electron micrographs of silk nanoparticles. (**A**) Pure *Ap*F nanoparticles; (**B**) DOX-*Ap*F nanoparticles; (**C**) Ibuprofen-*Ap*F nanoparticles; and (**D**) Ibuprofen-Na-*Ap*F nanoparticles. The fibroin concentration was 10 mg/mL. Scale bar = 10 µm.

Scanning electron micrographs (SEM) were used to evaluate the size, shape, and morphology of nanoparticles. Figure 1 shows SEM images of pure *Ap*F nanoparticles and drug-loaded *Ap*F nanoparticles. The pure *A*pF nanoparticles were spherical with a diameter of approximately 500 nm. When *Ap*F nanoparticles were loaded with small-molecule drugs, such as doxorubicin hydrochloride (DOX), ibuprofen, and ibuprofen-Na, the size and morphology of the drugs loaded on nanoparticles were similar to that of pure *Ap*F nanoparticles.

### 2.2. The Average Size and Brunauer-Emmett-Teller (BET) Surface Area of Particles

Particle size analysis was used to evaluate the quality of the nanoparticles. The average particle size of *Ap*F nanoparticles and drug-*Ap*F nanoparticles obtained using a Nano-ZS90 particle size analyzer are summarized in Table 1. The average size of pure *Ap*F nanoparticle was 496 ± 53.45 nm. After loading with small-molecule drugs (doxorubicin hydrochloride (DOX), ibuprofen, and ibuprofen-Na), the average particle size of the drugs loaded in nanoparticles were similar to that of pure *Ap*F nanoparticles. The results are consistent with the SEM data. The BET surface area of pure *Ap*F nanoparticles was 38.95 m^2^/g (Table 1). When *Ap*F nanoparticles were loaded with small-molecule drugs, the BET surface area decreased (DOX-A*p*F nanoparticles: 10.64 m^2^/g, ibuprofen-*Ap*F nanoparticles: 19.32 m^2^/g, and ibuprofen-Na-*Ap*F nanoparticles: 32.96 m^2^/g), which indicated the drugs has been successfully loaded onto *Ap*F nanoparticles.

### 2.3. Drug Loading

**Figure 2:**
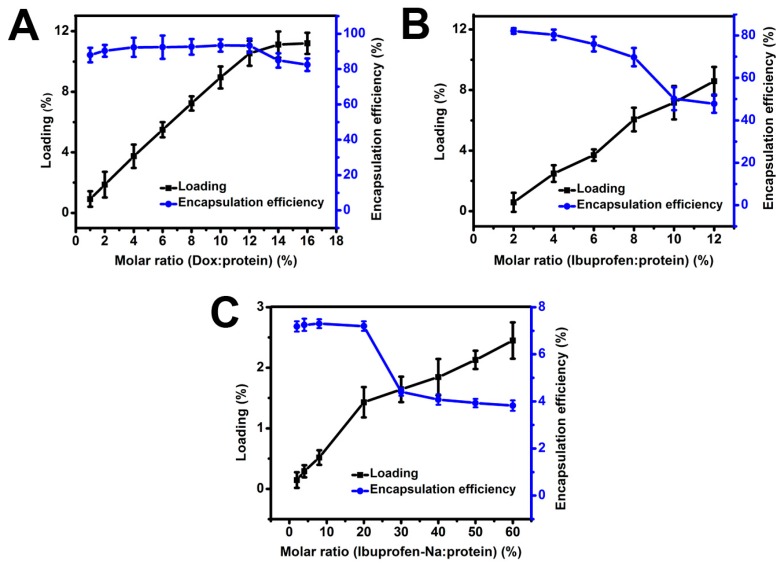
Drug loading and encapsulation efficiency of *Ap*F nanoparticles. (**A**) Doxorubicin (**B**) Ibuprofen and (**C**) Ibuprofen-Na loaded in *Ap*F nanoparticles.

In order to investigate the applicability of *Ap*F nanoparticles as a drug delivery system, three small-molecule model drugs with different charges—DOX (positive charge), ibuprofen (neutral charge), and ibuprofen-Na (negative charge)—were loaded on net negatively-charged silk fibroin nanoparticles by charge-charge interactions. The loading was examined with respect to the molar ratio of the model drug to *Ap*F (Figure 2A–C). As shown in Figure 2, there was a non-linear increase in the loading and encapsulation efficiency as greater quantities of model drug was added. Encapsulation efficiency above 93% was achieved at 10.5% loading for positively-charged DOX. After 10.5% loading, the encapsulation efficiency decreased, indicating that the protein matrix was saturated. Interestingly, when the non-ionic ibuprofen and the negatively-charged ibuprofen-Na were loaded on *Ap*F nanoparticles, the loading was low compared with DOX-*Ap*F systems. It may be that positively-charged DOX drug via electrostatic attraction (the zeta potential for the *Ap*F nanoparticles were measured as −23.8 mV), resulting in a high loading efficiency. However, the non-ionic ibuprofen and the negatively-charged ibuprofen-Na have weak interaction with *Ap*F nanoparticles possibly leads to lower binding than that of DOX. It may be that due to the weaker binding of ibuprofen-Na to *Ap*F materials, most ibuprofen-Na was extracted during *Ap*F nanoparticles preparation. Similar results can be concluded by Lammel and Wang et al. It is reported that loading and release of model drugs happens mostly through electrostatic interactions [28] and positively-charged drugs have better interactions with silk compared to negatively-charged drug [36].

### 2.4. Zeta Potential of Drug-Loaded ApF Nanoparticles

**Figure 3:**
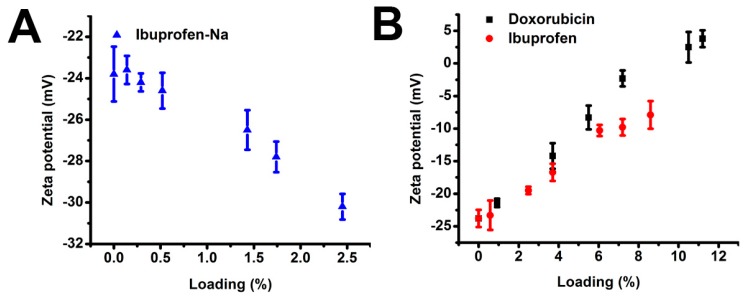
Zeta potential of (**A**) ibuprofen-Na and (**B**) doxorubicin and ibuprofen loaded on *Ap*F nanoparticles.

To better understand different drug loading, surface charges of the *Ap*F nanoparticles were determined. Changes in ζ-potential (mV) of *Ap*F nanoparticles loaded with drugs were evaluated at different loading percentages (Figure 3A,B). The ζ-potential of ibuprofen-Na-loaded nanoparticles was dependent on loading and increased with an increase in loading (Figure 3A). Briefly, the ζ-potential values of particles became more negative with increased loading. The ζ-potential values of pure *Ap*F nanoparticles and ibuprofen-Na-*Ap*F at an of 2.5% were −23.8 mV and −30.2 mV, respectively. Interestingly, the ζ-potential of DOX- and ibuprofen-loaded *Ap*F nanoparticles gradually decreased with increases in loading. At the same time, the ζ-potential of DOX became positive when loading exceeded 11% (Figure 3B), approximately the same point that the loading efficiency of DOX decreased (Figure 2A). This result indicates that the negative surface charge of the *Ap*F nanoparticles enables positively-charged small molecules to load by simple charge-charge interaction between the drug molecules. The silk nanoparticles were also compared with nanoparticle systems loaded with the non-ionic (ibuprofen) and negatively-charged (ibuprofen-Na) drugs (Figure 3).

### 2.5. Drugs Release Rate

**Figure 4:**
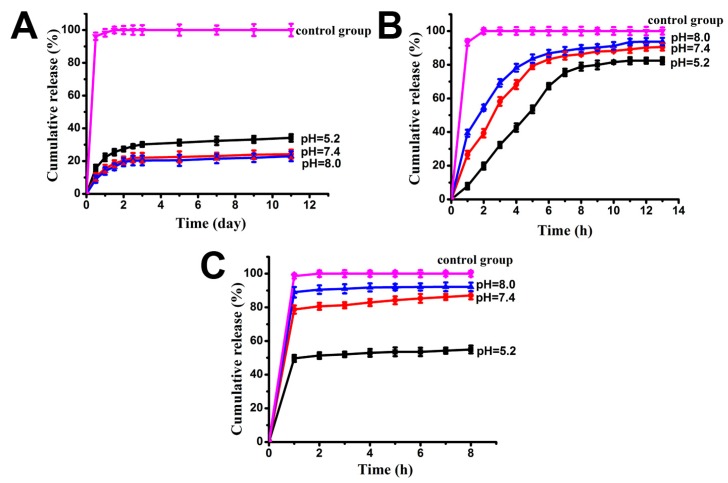
Cumulative release of model drugs from *Ap*F nanopartcles. (**A**) Positively-charged DOX from DOX-*Ap*F nanoparticles observed for a period of 11 days [40]; (**B**) non-ionic ibuprofen from ibuprofen-*Ap*F nanoparticles observed for 13 h; and (**C**) negatively-charged ibuprofen-Na from ibuprofen-Na-*Ap*F nanoparticles observed for 8 h. Equivalent free drugs (DOX, ibuprofen, ibuprofen-Na) were dispersed in PBS buffer as a control. Samples were in PBS solution at pH 5.2, 7.4, and 8.0 at 37 °C.

The release rate of drug-loaded *Ap*F is pH dependent (Figure 4). The cumulative release of DOX increased as pH decreased (Figure 4A). The release of DOX reached a maximum of 34.15% after 11 days at pH 5.2, which was higher than the release observed at pH 7.4 (24.23%) and 8.0 (22.96%). The approximate pKa of the doxorubicin amino group was 7.6 (37 °C, ionic strength 0.15) [42]. So doxorubicin hydrochloride will keep a positive charge in neutral and alkaline aqueous solutions. Some doxorubicin hydrochloride will change to the uncharged neutral molecule when the release solution changes to an acidic solution. This is why doxorubicin hydrochloride released quickly in the acidic solution than in the neutral and alkaline solutions. pH-dependent release is favorable in the therapeutic delivery of drugs, such as anti-cancer drugs. A lower pH environment is favored for the growth of tumor cells, whereas the opposite is true for normal cells [43].

However, the release of ibuprofen showed the opposite pattern (Figure 4B,C). There is a low-level and short initial burst release, and the reason may be that the residual ibuprofen drug was released from the surface of *Ap*F nanoparticles. The DOX and ibuprofen were released slowly. In contrast, ibuprofen-Na was released much faster, with more than 60% of total loading being released within one hour at nearly a zero-order release rate. It may be that negatively charged Ibuprofen-Na has weak interaction with *Ap*F nanoparticles, which lead to the drug molecule diffusion from nanoparticles. pKa values of ibuprofen is about 4.4 [44]. That is to say ibuprofen is an uncharged neutral molecule when it is in neutral aqueous solution or acidic solution. Only when the pH value was higher than 10 will it change to being negatively charged. In our release experiments, it will remain uncharged. Otherwise, for ibuprofen-Na, it will remain negatively charged during release experiments.

In addition, the cumulative release at pH 7.4 within the first 6 h was 85% for negatively-charged ibuprofen-Na, 83% for ibuprofen, and 10.3% for positively-charged DOX. This suggests that positively-charged molecules exhibit a more prolonged or sustained in vitro release of the drugs from the nanoparticles. This effect may be attributable to charge-charge interactions between the drug and the silk. There was a cumulative release of 100% of drugs in the control group. This result indicates that *Ap*F nanoparticles may be a suitable candidate for drug delivery.

### 2.6. Structure of Drug-ApF Nanoparticles

**Figure 5:**
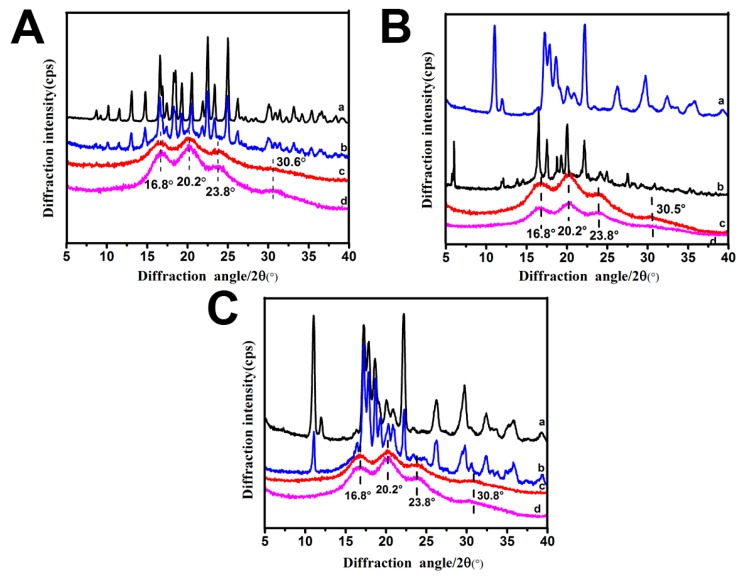
The structure of *Ap*F nanoparticles. (**A**) XRD of DOX-*Ap*F nanoparticles (**a**, DOX; **b**, DOX mixture with *Ap*F nanoparticles; **c**, DOX-*Ap*F nanoparticles; **d**, *Ap*F nanoparticles); (**B**) XRD of ibuprofen-*Ap*F nanoparticles (**a**, ibuprofen; **b**, ibuprofen mixture with *Ap*F nanoparticles; **c**, ibuprofen-*Ap*F nanoparticles; **d**, *Ap*F nanoparticles); (**C**) XRD of ibuprofen-Na-*Ap*F nanoparticles (**a**, ibuprofen-Na; **b**, ibuprofen-Na mixture with *Ap*F nanoparticles; **c**, ibuprofen-Na-*Ap*F nanoparticles; **d**, *Ap*F nanoparticles).

To confirm the changes in secondary structure between *Ap*F nanoparticles and drug-*Ap*F nanoparticles, X-ray diffraction curves were obtained (Figure 5). The pure *Ap*F nanoparticles (Figure 5A (curve d)) showed an X-ray diffraction profile with one intense diffraction peak at 20.2°, two minor peaks at 16.8° and 23.8°, and one weak peak at 30.8°. These peaks are typical of β-sheet conformation [45]. The DOX-*Ap*F nanoparticles (Figure 5A (curve c)) showed none of DOX’s characteristic diffraction peaks on the X-ray diffraction curve (Figure 5A (curve a)). In contrast, the mixture of DOX with *Ap*F nanoparticles (Figure 5A (curve b)) showed clear diffraction peaks that are characteristic of DOX (Figure 5A (curve a)). This observation indicates that there is no crystallization of DOX molecules in the nanoparticles. Similar changes were also observed in the ibuprofen-*Ap*F nanoparticle and ibuprofen-Na-*Ap*F nanoparticle systems (Figure 5B,C).

### 2.7. In Vitro Degradation of ApF Nanoparticles

**Figure 6:**
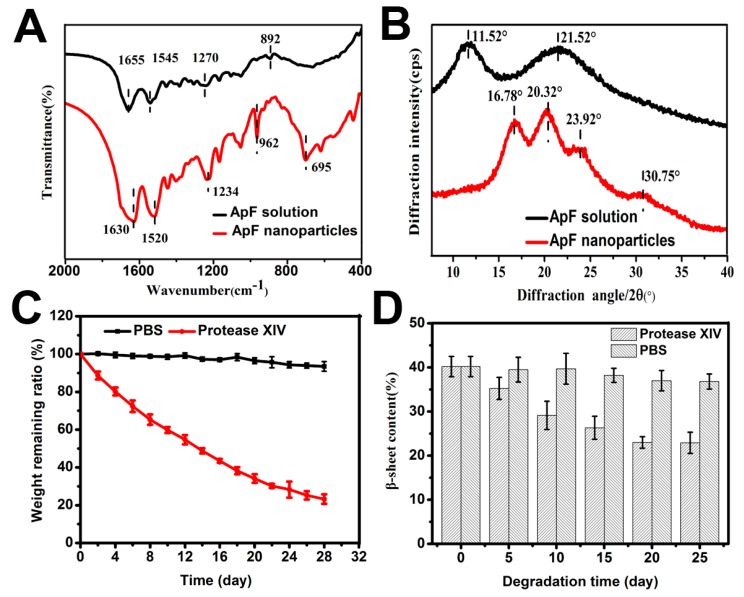
The secondary structure of *Ap*F nanoparticles and in vitro degradation of *Ap*F nanoparticles. (**A**) FTIR spectra of *Ap*F; (**B**) XRD of *Ap*F; (**C**) In vitro degradation of *Ap*F nanoparticles shown as percentage degradation over four weeks; and the (**D**) β-sheet content of silk crystals as a function of enzymatic degradation time by protease XIV and PBS denotes the data for the controls, treated by phosphate-buffered solution without enzyme.

It is reported that silk degradation is greatly affected by β-sheet formation [13]. To confirm the changes in the secondary structure during the formation of *Ap*F nanoparticles, FTIR spectra and X-ray diffraction curves were obtained. A freshly-prepared regenerated *Ap*F solution exhibited absorption bands at 1655 cm^−1^ (amide I), 1545 cm^−1^ (amide II), 1270 cm^−1^ (amide III), and 892 cm^−1^ (amide IV), assigned to an α-helix and random coil conformation [46]. In contrast, significant absorption bands at 1630 cm^−1^ (amide I), 1520 cm^−1^ (amide II), 1234 cm^−1^ (amide III), and 963 cm^−1^ (amide IV) appeared in *Ap*F nanoparticles (Figure 6A). These absorption bands are characteristic of the β-sheet structure [45], and indicate that a transformation from random coil and α-helix to β-sheet occurs in the process of *Ap*F nanoparticle preparation. As shown in the X-ray diffraction curves (Figure 6B), freshly-prepared regenerated *Ap*F solution exhibited two major diffraction peaks at 11.52° and 21.53°, corresponding to the α-helix structure [45]. Two intense diffraction peak at 16.78 and 20.32, and two minor peaks at 16.78 and 30.75, occurred in *Ap*F nanoparticles. These absorption bands are characteristic of the β-sheet conformation [45], and confirm that the *Ap*F sol-particle transition was accompanied by the *Ap*F conformational transformation, In accordance with the results from the FTIR spectra.

Biodegradability is one of the ideal properties of biomaterials used in tissue engineering. In in vitro degradation experiments, protease XIV is the most widely used proteolytic enzymes, which were derived from *Streptomyces griseus* [47,48]. Degradation of the *Ap*F nanoparticle pattern was determined based on the weight remaining ratio at different time points (2–28 days). After 28 days of degradation, there was a distinct change in the molecular weight of *Ap*F nanoparticles in protease XIV solution. The molecular weight of the nanoparticles reduced by 45.4% after 12 days. After 20 and 28 days, the molecular weight loss was approximately 65.9% and 86.8%, respectively. The remaining quantity of *Ap*F nanoparticles was approximately 23.2% after 28 days of degradation (Figure 6C). Conversely, the change of *Ap*F nanoparticles in the PBS solution was negligible in comparison to the change in the nanoparticles in the protease XIV solution. The remaining quantity of nanoparticles in the control group was 93.4% after 28 days. It is reported that the degradation of silk significantly depends on the molecular weight, the amount of crystalline structure, and structural characteristics, such as surface roughness, porosity, and pore size [13]. To reveal the degradation of *Ap*F nanoparticles structures by the proteolytic enzymes, the percentages of β-sheet structures of *Ap*F nanoparticles at various enzymatic degradation time by protease XIV and PBS were calculated based on the experimentally-obtained curves, as shown in Figure 6D. From control experiments without proteolytic enzyme for 25 days, the average β-sheet content decreased from 40% to 36%. However, treatment for 20 days by protease XIV, the β-sheet content reduced to 23%. After enzymatic degradation over 20 days, the β-sheet content of the *Ap*F crystals did not change at these levels. The result indicates that 23% of the β-sheet structure still existed in *Ap*F crystals, and then the crystalline structure collapsed into small fragments, during longer enzymatic degradation time by protease XIV.

### 2.8. Cell Viability

**Figure 7:**
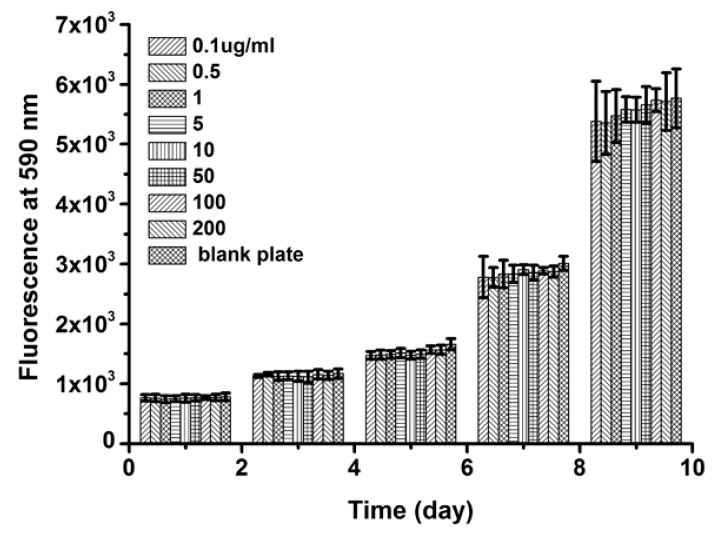
The cellular activity was detected by Alamar Blue assay after being treated with different concentrations of *Antheraea pernyi* fibroin nanoparticles for 1–9 days. The concentration of *Ap*F nanoparticles was 200, 100, 50, 10, 5, 1, 0.5, and 0.1 µg/mL. The blank plate was the control group.

The Alamar Blue assay was carried out using the L929 cells to research the cell viability (Figure 7). As culture days became longer, the cell activity gradually increased on all the samples. Nevertheless, there were no significant differences for the relative cells activity between the different concentrations of *Ap*F nanoparticles (200, 100, 50, 10, 5, 1, 0.5, and 0.1 µg/mL) and the blank plate at the same culture time (1, 3, 5, 7, and 9 days). These results implied that the concentration of *Ap*F nanoparticles had no significant effects on the cells activity. Therefore, the *Ap*F nanoparticles were suitable for cell proliferation.

**Figure 8:**
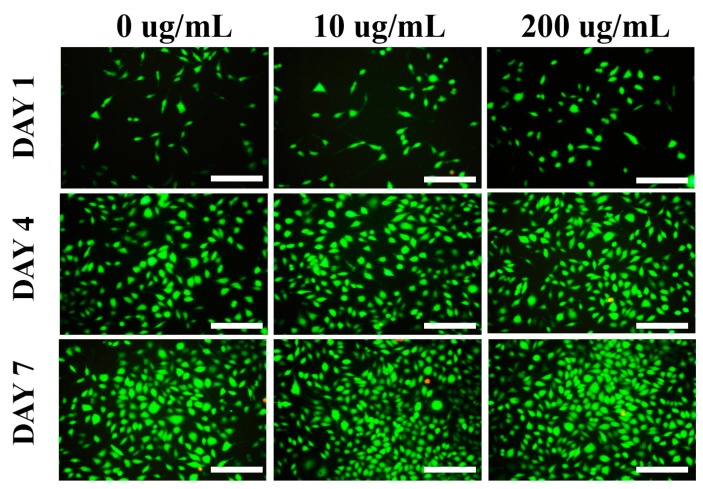
Fluorescence images of L929 cells cultured in the medium with different concentrations of *Ap*F nanoparticles (10 and 200 µg/mL) and blank plate (0 µg/mL). Green fluorescence represents the live cells and red fluorescence dots indicate the dead cells. Scale bar = 200 µm.

The L929 cells were cultured in the medium with different concentrations of *Ap*F nanoparticles. Green fluorescence represents the live cells and red fluorescence dots indicate the dead cells. It was observed that the live cells (green) attached well and grew normally in the medium with different concentrations of *Ap*F nanoparticles (10 and 200 µg/mL) and the blank plate (Figure 8) as well and, from day three, onward, the cells began to proliferate more quickly. With the increase of incubation times, more live cells were detected in all groups. Furthermore, very few or no dead cells were detected from these scaffolds. These results coincided with the result of the Alamar Blue assay. These observation indicates that the *Ap*F nanoparticles possess good biocompatibility and support the growth of cells.

## 3. Discussion

As a potential biomaterial, silk fibroin is used as a platform to enhance cell adhesion and proliferation and differentiation [21,49]. Although much research has been done on mulberry silk investigating its potentials as a biomaterial, the work done on non-mulberry silk is still limited. Non-mulberry silk can be a potential biomaterial, since it has superior mechanical properties compared to mulberry silk and is also characterized by the presence of RGD sequences [21,22,23]. Therefore, the exploitation of the biomaterial properties of non-mulberry silk fibroin will open a new era of an alternative natural functional biomaterial to replace mulberry silk protein fibroin. In order to evaluate the potential of non-mulberry silk-based biomaterials in various biomedical applications such as tissue engineering and as a model matrix to study various cellular phenomena, numerous studies have been carried out. Non-mulberry silk has also been exploited for fabricating drug delivery devices in the form of nanoparticles from fibroin. It is reported that silk fibroin nanoparticles of non-mulberry *A. mylitta* for the delivery of anti-cancer therapeutics have been studied [50], which indicated the potential of non-mulberry silk as a delivery vehicle. *Antheraea pernyi* is one of the most well-known wild non-mulberry silk sources and also contains an integrin-binding RGD peptide [23], which is known as a recognition motif in several different integrin receptors [51,52,53,54]. Notably, RGD peptide has been applied widely in drug delivery [55,56,57]. It is reported that RGD-containing materials may specifically target drugs to cancer cells or angiogenic endothelial cells by the binding of the RGD peptide to these cell surface receptors. Moreover, these materials can be internalized by receptor-mediated endocytosis. This is an advantage compared with many other non-RGD materials. Therefore, choosing non-mulberry-*Antheraea pernyi* silk as a platform for drug delivery is justified.

Silk fibroin is increasingly being considered as a suitable protein based material for drug delivery applications [58,59,60,61]. Silk protein-based nanoparticles also exhibit superior performance for sustained release of drugs and genes [62,63]. The mechanism of binding drugs to silk fibroin is an important consideration for drug delivery. Loading and release of model drugs of various molecular weights and surface charges have been widely studied [31]. It is reported that loading and release of model drugs happens mostly through electrostatic interactions [28]. Strong electrostatic binding between silk and bound molecules can avoid significant burst release [64]. Though such strong interactions may also prevent complete release of the carried molecules, the release can be controlled by adjusting the surrounding charge by changing the pH. In addition, drug transfer can be controlled by adjusting the composition/structure of the silk coating [65,66]. In addition to release from the intact particle, drugs can also be released when the particles are enzymatically degraded. The microstructure of silk plays an important role in both drug release and particle degradation. The microstructure of silk can be controlled by inducing β-sheet formation during particle regeneration from solution. It is reported that an increase in β-sheet content is responsible for slowing down the release rate [29]. The methods of silk particles preparation have been widely studied for drug delivery applications. A mild environment, such as aqueous solution and ambient temperature is needed to load the model drugs during the particle fabrication process. The present study provides a unique method to fabricate *Ap*F nanoparticles and *Ap*F nanoparticles were used as drug carriers to load differently-charged small-molecule drugs via simple absorption based on electrostatic interactions under mild conditions. This method avoids using organic solvents and any other noxious reagents during material processing. Therefore, this method is suitable for biomedical applications and *Ap*F nanoparticles have potential as a sustained drug delivery vehicle.

## 4. Materials and Methods

Chinese tussah silkworm, *Antheraea pernyi*, silk cocoons were obtained from Liaoning Province (Liaoning Meilin Group Co., Ltd., Liaoning, China). All reagents, unless stated otherwise, were purchased from Sigma-Aldrich (St. Louis, MO, USA). 

### 4.1. Preparation of ApF Solution

The *Antheraea pernyi* silk fibroin (*Ap*F) was prepared as an earlier published procedure. Briefly, cocoons of *Antheraea pernyi* were boiled for 30 min in 0.2% sodium bicarbonate solution at 100 °C to remove the sericin. After being rinsed and dried, the degummed silk fibroin was dissolved in 9 M LiSCN solution, and then dialyzed against distilled water for four days to obtain a pure fibroin solution [25]. As determined gravimetrically, the final silk fibroin concentration was approximately 2.2% (*w*/*v*) and later diluted to the desired concentration.

### 4.2. Preparation of ApF Nanoparticles

*Ap*F nanoparticles were fabricated by ion induction. Briefly, the 10 mg/mL *Ap*F solution was mixed with 1 mM Ca^2+^ in 1:1 volume ratios using a pipette. The mixture was placed in a water bath for 60 min at a temperature 37 °C to induce self-assembly. In order to get pure *Ap*F nanoparticles, the resulting suspension was centrifuged at 12,000 rpm for 10 min and then washed three times using ultrapure water.

### 4.3. Drug Loading in ApF Nanoparticles

Drug loading on the silk particles was conducted as follows: small-molecule model drugs with different charges (doxorubicin hydrochloride, ibuprofen (dissolved with acetone), and ibuprofen-Na) were dissolved in 10 mL of an aqueous solution of 1 mM Ca^2+^. Approximately 10 mg/mL *Ap*F solution was added to the drug solution at a 1:1 volume ratio containing different molar ratios to prepare the drug-encapsulated nanoparticles. The mixture was placed in a water bath for 60 min at a temperature 37 °C to induce self-assembly. To obtained pure drug-loaded *Ap*F nanoparticles, the suspension was centrifuged at 12,000 rpm for 10 min and then washed three times using ultrapure water to remove the excess drug molecules. In order to determine drug loading and encapsulation efficiency, the supernatants were subjected to absorbance measurements using a UV-VIS absorption spectrophotometer (Bio-Rad, Berkeley, CA, USA). The drug quantification was calculated based on a standard calibration curves. The model drugs were dispersed in 10 mL water as the control for each experiment. Finally, Drug loading and encapsulation efficiency were calculated via Equations (1) and (2), respectively:

(1) $$\text{Drug loading}\left(w/w\%\right)=\frac{\text{amount of model drug in particles}}{\text{model drug of particles}}\times 100$$

(2) $$\text{Encapsulation Efficiency}\left(w/w\%\right)=\frac{\text{amount of drug in particles}}{\text{drug added}}\times 100$$

### 4.4. Zeta Potential and Surface Area Analysis

To investigate the influence of *Ap*F nanoparticle loading, surface charges of the particles were measured using a zeta potential analyzer (Malvern Instruments, Malvin, UK). The surface areas of particles were measured by Brunauer-Emmett-Teller (BET) methods using nitrogen adsorption-desorption measurements in a V-Sorb 2800P (Micromeritics, Norcross, GA, USA) surface area analyzer.

### 4.5. Release of Drugs from ApF Nanoparticles

Ten milligrams of the drug-loaded *Ap*F particles were subsequently re-dispersed in 10 mL of phosphate-buffered saline (PBS) buffer with pH values of 5.2, 7.4, and 8.0 at 37 °C to monitor the pH-dependent release and free drugs were dispersed in PBS buffer as the control. At pre-determined time points, the samples were centrifuged at 12,000 rpm for 10 min to collect the supernatants. Next, the nanoparticles were suspended in fresh PBS buffer to continue the release study. The supernatants were subjected to absorbance measurement using a UV-VIS absorption spectrophotometer. The drug content in the medium was calculated based on a standard calibration curves. All measurements were performed in triplicate. The percentage of cumulative model drug release (%*w*/*w*) was calculated using Equation (3):

(3) $$\text{Releasing content}\left(w/w\%\right)=\frac{\text{amount of drug in the release medium}}{\text{amount of drug loaded into particle}}\times 100$$

### 4.6. Morphology and Particle Size of Drug-Loaded ApF Nanoparticles

The morphology of pure *Ap*F nanoparticles and drug-loaded *Ap*F nanoparticles was examined via scanning electron microscopy (SEM, Hitachi S-4700, Tokyo, Japan) at an accelerating voltage of 15 kV. *Ap*F nanoparticles were distributed in water by ultra-sonication, plated directly on a silicon plate, and later dried by vacuum. The samples were gold sputter-coated to prevent charging during SEM imaging. To better understand different drug-loaded *Ap*F nanoparticle sizes, the samples were carried out using a NanoZS90 particle size analyzer (Malvern Instruments, Malvin, UK).

### 4.7. Structure of ApF Nanoparticles

*Ap*F solutions, pure *Ap*F nanoparticles and drug-loaded *Ap*F nanoparticles were frozen at −80 °C and subsequently freeze-dried for X-ray diffraction (XRD) analysis. XRD analysis was conducted on an X’PERT-PRO MPD Diffractometer (Panalytical Co., Almelo, The Netherlands) with a Cu-Kα radiation source. The scanning speed was 2°/min. The X-ray source was operated at a 30 kV voltage and 20 mA current. XRD patterns were recorded in the 2θ region from 5° to 40°. In addition, the FTIR spectra of *Ap*F solutions and *Ap*F nanoparticles were obtained using a Nicolet 5700 Fourier transform infrared spectrometer (Nicolet Co., Madison, WI, USA) in the spectral region of 400–4000 cm^−1^.

### 4.8. In Vitro Biodegradation of ApF Nanoparticles

One hundred milligrams of nanoparticles were separately placed into a tube containing 10 mL of a PBS solution with protease XIV (pH 7.4, 37 °C, 5 U/mL). A similar sample was kept in PBS as a control. The protease XIV solution was replaced every two days with freshly prepared solution. The samples were centrifuged at 12,000 rpm for 5 min at pre-determined time points (2, 4, 6, 8, 10, 12, 14, 16, and 18 days). The pellets were lyophilized to obtain data on degraded *Ap*F nanoparticles, if any. The change in the weight remaining ratio was calculated using Equation (4):

(4) $$\text{Remain weight}\left(\%\right)=\frac{\text{amount of nanoparticles}-\text{amount of degradation}}{\text{amount of nanoparticles}}\times 100$$

### 4.9. Cell Culture

L929 cells were maintained in Dulbecco’s Modified Eagle’s Medium (DMEM; Gibco, Invitrogen, Carlsbad, CA, USA) containing 10% fetal bovine serum (FBS; Gibco, Invitrogen), and 1% penicillin-streptomycin solution at 37 °C in a 5% CO_2_ humidified atmosphere. The medium was changed every 2–3 days.

The L929 cells were plated at a density of 7 × 10^3^ cells/well in 96-well plates at 37 °C in a 5% CO_2_ atmosphere. After 24 h of culture, the medium in the well was replaced with 100 µL fresh medium containing *A. pernyi* silk fibroin nanoparticles of varying concentrations (200, 100, 50, 10, 5, 1, 0.5, or 0.1 µg/mL) and incubated for a pre-determined time interval (1, 3, 5, 7, or 9 days). A blank plate was used as the control group. The medium was replaced with fresh medium containing *A. pernyi* silk fibroin nanoparticles of varying concentrations every three days. After subsequent incubation, the cell proliferation was evaluated with Alamar Blue (AB) assay and all experiments were performed in triplicate. After cell culture for 1, 4, and 7 days, the cytotoxicity of the treated cells (200, 10, and 0 µg/mL) were examined with live/dead assay (Calcein AM and PI) under inverted fluorescence microscopy (Olympus Corporation, Tokyo, Japan).

### 4.10. Statistical Analysis

Statistical analysis was performed using one-way ANOVA. The data were presented as the mean ± SD. *p*-Values < 0.05 were considered to be statistically significant.

## 5. Conclusions

We show the applicability of nanoparticles from the non-mulberry silkworm *Antheraea pernyi* for controlled drug release. The negative surface charge of the fibroin nanoparticles enables loading with different types of charged small molecules by charge-charge interaction and diffusion into the particle matrix. In vitro release reveals that the release of small molecules depends on their charge interactions between the drugs and the silk fibroin. In addition, the release rate of loaded drugs from fibroin nanoparticles is observed to be pH-sensitive. The biodegradable behavior, cell viability, and growth, and simplicity of the all-aqueous production and loading process suggest that the fibroin nanoparticles of this underutilized can be exploited as an alternative matrix for drug carrying and controlled release in diverse biomedical applications.

## Figures and Tables

**Table 1:** The average size and BET surface area of *Ap*F nanoparticles and drug- *Ap*F nanoparticles.

Samples	The Average Particle Size (nm)	BET Surface Area (m^2^/g)
*Ap*F nanoparticles	496 ± 53.45	38.95
DOX-*Ap*F nanoparticles	522 ± 62.63	10.64
Ibuprofen-*Ap*F nanoparticles	504 ± 48.43	19.32
Ibuprofen-Na-*Ap*F nanoparticles	519 ± 36.98	32.96
